# Pseudohyperkalemia as a New Insight Into the Causes of Hyperkalemia Associated With Atopic Dermatitis

**DOI:** 10.7759/cureus.79943

**Published:** 2025-03-03

**Authors:** Atsuhiro Yae, Junji Azuma, Eri Kijima, Takehisa Yamamoto, Yasuhiro Hasegawa

**Affiliations:** 1 Pediatrics, Minoh City Hospital, Minoh, JPN

**Keywords:** atopic dermatitis, hyperkalemia, platelet count, potassium, pseudohyperkalemia, thrombocytosis

## Abstract

Pseudohyperkalemia is defined as a falsely elevated serum potassium level caused by the release of potassium from blood cells during the clotting process, which occurs in vitro. Although hyperkalemia, a potentially life-threatening condition, has been associated with severe atopic dermatitis (AD) through mechanisms such as the 'aldosterone paradox,' pseudohyperkalemia has not been documented in AD. We present a case of pseudohyperkalemia in a nine-month-old girl with thrombocytosis associated with severe AD. Despite a serum potassium level of 7.3 mEq/L, the whole-blood potassium measured using a blood gas analyzer was 4.8 mEq/L. Therefore, we diagnosed the case as pseudohyperkalemia and decided not to treat the serum hyperkalemia. Following treatment with topical steroids and a Janus kinase inhibitor, the pseudohyperkalemia resolved as the skin condition improved and the platelet count decreased.

## Introduction

Hyperkalemia, which can lead to life-threatening arrhythmias, is a medical emergency requiring urgent treatment. Conversely, pseudohyperkalemia refers to a falsely elevated potassium concentration in blood samples measured in vitro, while the actual (in vivo) potassium levels remain within the normal range. This condition does not require treatment. Furthermore, electrocardiogram (ECG) changes, generalized muscle weakness, or paralysis associated with hyperkalemia are not observed [1,2]. Pseudohyperkalemia occurs when potassium is released from the blood cells during or after blood collection, often due to issues with sample handling or specific patient conditions, including thrombocytosis, leukocytosis, and inherited defects in red blood cell membrane structure [1,2].

Atopic dermatitis (AD) is a chronic, pruritic, recurrent inflammatory skin disease that may result in thrombocytosis [3]. There have been several documented cases of hyperkalemia in AD, which may be related to the so-called 'aldosterone paradox' [4,5]. However, pseudohyperkalemia has not been previously documented in this condition. Here, we present a case of pseudohyperkalemia in an infant with thrombocytosis secondary to severe AD.

## Case presentation

The case involves a nine-month-old female child who was born at 40 weeks and 4 days and weighed 3,714 g at birth. There were no complications in her perinatal history, and she was born without neonatal asphyxia or other complications. At around one month of age, she developed rashes all over her body and was prescribed topical steroids by her family doctor. However, due to family preference, the use of topical steroids was discontinued, leading to poor control of the rash. She was fed a combination of breastmilk and formula; however, at five months of age, the mother switched to exclusive breastfeeding. At nine months of age, she was referred to our hospital because of poor weight gain and developmental delay. Her height was 67.4 cm (−0.6 SD) and her weight was 6.5 kg (−1.8 SD). There were delays of approximately five months in gross motor, language, and fine motor development, as assessed using the Denver Developmental Screening Test. The family history revealed a shellfish allergy in the father and childhood asthma in the mother.

**Figure 1:**
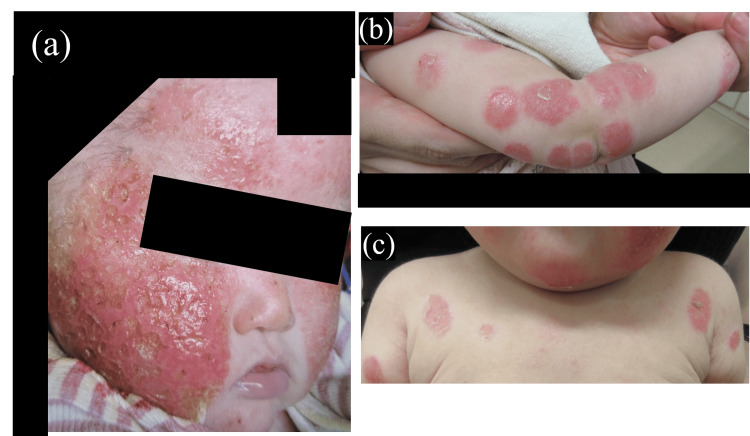
Eczema on both the cheeks and around the mouth (a), and erythematous circular rashes on the limbs and the anterior chest (b, c)

Skin examination revealed eczema on both the cheeks and around the mouth, along with erythematous circular rashes on the limbs and the anterior chest (Figure 1).

Brain magnetic resonance imaging revealed no abnormalities. Laboratory data at admission (day 1) revealed a white blood cell count of 26,000 /µL with 25.3% eosinophils, a platelet count of 80.5 × 10^4^ /µL, a protein level of 5.3 g/dL, an albumin level of 3.6 g/dL, and serum sodium level of 117 mEq/L. The serum potassium level was elevated to 7.3 mEq/L (Table 1). Other findings included a zinc level of 67 µg/dL, cortisol level of 11 ug/dL, renin level of 142.3 pg/mL, aldosterone level of 836 pg/mL, immunoglobulin G level of 182 mg/dL, nonspecific immunoglobulin E (IgE) level of 5,380 IU/mL, and high thymus and activation-regulated chemokine level of 3,732 pg/mL (Table 1).

**Table 1:** Trends in the laboratory results

Parameter	Units	Day 1	Day 5	Day 15	Reference range
White blood cell count	/μL	26,000	21,900	14,000	6,000-17,500
Neutrophils	%	13.7	18.9	23.5	39-73
Eosinophils	%	25.3	13.7	8.1	0-8
Red blood cell count	10^4^/μL	454	407	412	380-480
Hemoglobin	g/dL	12.6	11.3	11.9	12-15
Platelet	10^4^/μL	80.5	68.5	53.2	15-45
Total protein	g/dL	5.3	4.3	5.4	6.0-7.6
Albumin	g/dL	3.6	2.8	3.6	3.5-5.0
Sodium	mEq/L	117	136	138	135-145
Serum potassium	mEq/L	7.3	4.7	4	3.5-5.0
Whole-blood potassium	mEq/L	4.84	3.34	3.24	3.5-5.0
Chloride	mEq/L	94	107	108	98-108
Calcium	mg/dL	10.3	9.2	9.5	8.7-11.0
Blood urea nitrogen	mg/dL	13	2	3	2.5-7.0
Creatinine	mg/dL	0.34	0.24	0.27	0.14-0.31
Bilirubin total	mg/dL	0.19	<0.10	0.1	0.2-1.2
Aspartate aminotransferase	U/L	41	41	29	25-90
Alanine aminotransferase	U/L	22	28	12	10-55
Gamma-glutamyltransferase	U/L	18	13	17	10-60
Alkaline phosphatase	U/L	126	104	300	300-1200
Lactate dehydrogenase	U/L	237	310	247	200-500
C-reactive protein	mg/dL	<0.02	<0.02	<0.02	<0.29
Creatinine kinase	U/L	30	39	44	100-400
Adrenocorticotropic hormone	pg/mL	2	-	-	7.2-63.3
Cortisol	ug/dL	11	-	-	3.7-19.4
Renin	pg/mL	142.3	-	-	2.2-39.5
Aldosterone	pg/mL	836	-	-	50-150
Immunoglobulin G	mg/dL	182	-	-	360-1010
Immunoglobulin A	mg/dL	25	-	-	10-50
Immunoglobulin M	mg/dL	30	-	-	20-100
Nonspecific Immunoglobulin E	IU/mL	5,380	-	-	1-15
Thymus and activation-regulated chemokine	pg/mL	3,732	-	-	<1367
Zinc	µg/dL	67	-	-	70-120

**Figure 2:**
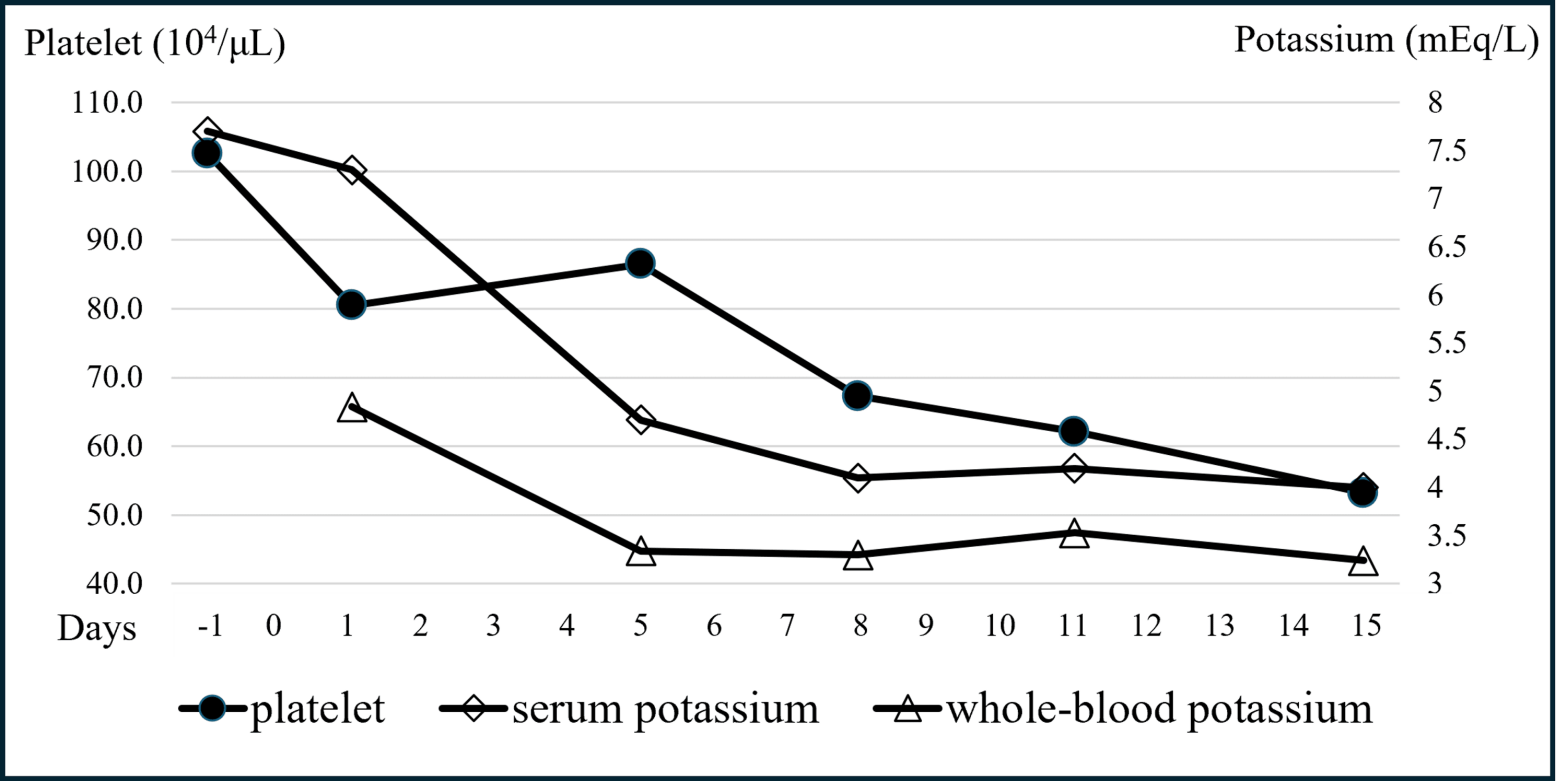
Platelet counts decreased over time, and the discrepancy between whole-blood and serum potassium levels was diminished

In contrast, the potassium level in an anticoagulated whole-blood sample, measured by a blood gas analyzer, was 4.8 mEq/L (Figure 2, Table 2).

**Table 2:** Venous blood gas analysis

Parameter	Units	Results	Reference range
pH	-	7.325	7.350-7.450
pCO2	mmHg	24.4	40-55
HCO3	mmol/L	12.4	18-26
Actual base excess	mmol/L	-12	-4.0-2.0
Sodium	mEq/L	127.5	135-145
Potassium	mEq/L	4.84	3.5-5.0

The allergen-specific IgE test revealed the presence of multiple food allergies, including egg white and ovomucoid (Class 6); egg yolk and beef (Class 5); milk, wheat, salmon roe, and ω-5 gliadin (Class 4); rice (Class 3); and chicken, cod, tuna, and soy (Class 2) (Table 3).

**Table 3:** Allergen-specific IgE tests

Allergen	Specific IgE (UA/mL)	Class
Egg white	>100	6
Egg yolk	57.2	5
Ovomucoid	>100	6
Milk	44.8	4
Beef	57.7	5
Chicken	2.09	2
Cod	2.83	2
Tuna	1.63	2
Wheat	35.8	4
Rice	3.89	3
Soybeans	1.37	2
Salmon roe	36.3	4
ω-5 gliadin	25.5	4

A 12-lead ECG showed no abnormalities in tented T waves. Based on these findings, the patient she was diagnosed with food allergies and severe AD complicated by impetigo contagiosa. She had a severe condition with an Eczema Area and Severity Index (EASI) score of 22.2. The patient was treated with topical steroids and intravenous cefotaxime, and a food allergy elimination diet was initiated. Despite a marked elevation in serum potassium, no electrocardiographic abnormalities were noted, and no generalized muscle weakness or paralysis was observed. Since the whole-blood potassium level was normal, we suspected pseudohyperkalemia associated with thrombocytosis and decided not to correct the serum potassium levels.

By the 14th day of hospitalization, her skin symptoms improved gradually with topical steroids and a topical Janus kinase inhibitor. As her skin symptoms decreased, her laboratory abnormalities also improved in parallel (Table 1). The sodium levels normalized without treatment, the platelet count decreased, and the discrepancy between the whole-blood and serum potassium levels diminished (Table 1, Figure 2). At two years of age, her skin condition was well-controlled with topical steroids and the food allergy elimination diet. A slight verbal developmental delay was observed, which is currently improving. An oral food challenge is planned in the future to assess further progress.

## Discussion

Pseudohyperkalemia was first described by Hartmann and Mellincoff in 1955 in patients with thrombocytosis [6]. These patients had high serum potassium levels but normal plasma potassium, leading to the initial understanding of the condition. Over time, the definition has expanded to encompass several mechanisms that cause false elevations of potassium levels in both serum and plasma laboratory tests [1]. The recognition of pseudohyperkalemia has emphasized the impact of specimen collection and handling practices, such as hemolysis, improper storage, and delays in processing, on test accuracy [1]. In addition, rare patient-specific factors, such as inherited red cell membrane disorders and hematological abnormalities, have been identified. Thrombocytosis, in particular, is a major cause of pseudohyperkalemia associated with hematological abnormalities [1,2,7].

Platelets have been shown to play a significant role in allergic inflammatory diseases [8,9], and thrombocytosis has been reported in several cases of AD [2,4,10]. Akcal et al. reported that patients with AD had a statistically higher mean platelet count compared to controls. Furthermore, platelet counts were significantly higher in patients with severe and moderate AD than those with mild AD [3]. While these findings suggest that AD may contribute to pseudohyperkalemia due to thrombocytosis, to the best of our knowledge, no reports of pseudohyperkalemia associated with AD have been reported. This suggests that pseudohyperkalemia could be a novel cause of serum hyperkalemia in AD.

Pseudohyperkalemia, associated with thrombocytosis, is caused by the release of potassium from numerous platelets during the clotting process in serum samples [1]. Consequently, this issue occurs only when serum is used to measure potassium, as anticoagulated samples remain unaffected, even with markedly elevated platelet counts [1,11]. Ranjitkar et al. suggested that patients with platelet counts >50 × 10^4^/µL are at increased risk of developing pseudohyperkalemia [11]. Consequently, in instances of serum hyperkalemia with platelet counts greater than 50 × 10^4^/µL, it is essential to assess potassium levels using plasma derived from anticoagulated blood samples or whole-blood samples that have undergone anticoagulation [1,11]. In clinical settings, measuring whole-blood potassium using a point-of-care blood gas analyzer is a rapid and effective method for confirming pseudohyperkalemia due to thrombocytosis [1,11]. In this case, the significant increase in platelet count upon admission likely contributed to the development of pseudohyperkalemia. However, by the seventh day, the platelet count had increased to 86 × 10^4 ^µL, but the discrepancy between the serum and whole-blood potassium levels had diminished. The serum potassium was 4.7 mEq/L, while the whole-blood potassium was 3.3 mEq/L (Figure 2). Consequently, it is essential to consider other potential causative factors, such as pseudohyperkalemia resulting from the sample handling practices mentioned earlier. The underlying causes of the residual differences between the serum and whole-blood potassium levels remain to be elucidated (Figure 2). However, Ranjitkar et al. documented that in the majority of cases across platelet counts, the potassium levels in serum samples were higher than those in whole blood [11]. One hypothesis posits that these discrepancies can be attributed to the release of potassium from platelets during the clotting process in the serum samples.

Adachi et al. conducted a comprehensive search of PubMed and the Igaku Chuo Zasshi (ICHUSHI) database maintained by the Japan Medical Abstracts Society [4]. The search terms used included “atopic dermatitis AND (hyperkalemia OR hyponatremia OR electrolyte imbalance OR pseudohypoaldosteronism).” The results of this extensive search revealed 36 cases of severe AD with hyponatremia, which were associated with a tendency to present with hyperkalemia, metabolic acidosis, and increased renin-angiotensin-aldosterone activity. The authors postulated that the primary cause of hyponatremia may be sodium loss in the exudates from the damaged skin, as the urinary sodium levels were low, and hyperkalemia may occur due to reduced sodium delivery to the distal nephron, which activates the 'aldosterone paradox' mechanism and inhibits potassium secretion [4]. In this case, we observed low levels of sodium, protein, and albumin, along with elevated renin and aldosterone levels, and slightly elevated whole-blood potassium. However, the serum hyperkalemia observed in this case was due to pseudohyperkalemia. It is hypothesized that some reported cases of hyperkalemia in severe AD may involve pseudohyperkalemia, as they were associated with markedly elevated platelet counts [4,5]. Since pseudohyperkalemia does not require treatment, and therapeutic intervention may induce hypokalemia, prompt diagnosis of this condition is essential. This ensures the patient does not receive inappropriate treatment. Consequently, pseudohyperkalemia should be considered in patients with AD who have elevated platelet counts and serum potassium levels.

This report is based on single-patient data. Therefore, future studies are needed to clarify the prevalence of thrombocytosis and pseudohyperkalemia in AD. Although the exact prevalence of thrombocytosis in AD remains uncertain, Adachi et al. reported that 37% of patients with severe AD had elevated platelet counts [4]. Akcal et al. also reported that patients with moderate or severe AD (n=51) had higher mean platelet counts (>57 × 10^4^/µL) than those with mild AD (n=116) [3]. Therefore, we suggest confirming thrombocytosis and potassium levels with anticoagulated blood samples in all patients with moderate or severe AD.

## Conclusions

In this case report, we describe pseudohyperkalemia associated with thrombocytosis in severe AD. Previously, the etiology of hyperkalemia in severe AD was thought to be linked to mechanisms such as the 'aldosterone paradox.' However, while chronic inflammation in AD leads to thrombocytosis, which may increase the risk of pseudohyperkalemia, no cases of pseudohyperkalemia have been reported in AD. This report is the first to identify pseudohyperkalemia as a potential cause of serum hyperkalemia in severe AD with thrombocytosis. Recognizing this phenomenon in moderate or severe AD is crucial to prevent inappropriate intervention. Future studies should focus on investigating the prevalence of thrombocytosis and pseudohyperkalemia in AD.

## References

[1] Meng QH, Wagar EA (2015). Pseudohyperkalemia: a new twist on an old phenomenon. Crit Rev Clin Lab Sci.

[2] Sevastos N, Theodossiades G, Archimandritis AJ (2008). Pseudohyperkalemia in serum: a new insight into an old phenomenon. Clin Med Res.

[3] Akcal O, Taskırdı İ (2022). Do platelet count and mean platelet volume have a predictive role as a marker in children with atopic dermatitis?. Indian J Dermatol.

[4] Adachi M, Takamasu T, Inuo C (2019). Hyponatremia secondary to severe atopic dermatitis in early infancy. Pediatr Int.

[5] Jo SY, Lee C-H, Jung W-J, Kim S-W, Hwang Y-H (2018). Common features of atopic dermatitis with hypoproteinemia. Korean J Pediatr.

[6] Hartmann RC, Auditore JV, Jackson DP (1958). Studies on thrombocytosis. I. Hyperkalemia due to release of potassium from platelets during coagulation. J Clin Invest.

[7] Sevastos N, Theodossiades G, Efstathiou S, Papatheodoridis GV, Manesis E, Archimandritis AJ (2006). Pseudohyperkalemia in serum: the phenomenon and its clinical magnitude. J Lab Clin Med.

[8] Page C, Pitchford S (2014). Platelets and allergic inflammation. Clin Exp Allergy.

[9] Pitchford SC (2007). Defining a role for platelets in allergic inflammation. Biochem Soc Trans.

[10] Nomura I, Katsunuma T, Tomikawa M (2002). Hypoproteinemia in severe childhood atopic dermatitis: a serious complication. Pediatr Allergy Immunol.

[11] Ranjitkar P, Greene DN, Baird GS, Hoofnagle AN, Mathias PC (2017). Establishing evidence-based thresholds and laboratory practices to reduce inappropriate treatment of pseudohyperkalemia. Clin Biochem.

